# Assessing Autosomal InDel Loci With Multiple Insertions or Deletions of Random DNA Sequences in Human Genome

**DOI:** 10.3389/fgene.2021.809815

**Published:** 2022-02-01

**Authors:** Yining Yao, Kuan Sun, Qinrui Yang, Zhihan Zhou, Chengchen Shao, Xiaoqin Qian, Qiqun Tang, Jianhui Xie

**Affiliations:** ^1^ Department of Forensic Medicine, School of Basic Medical Sciences, Fudan University, Shanghai, China; ^2^ Department of Biochemistry and Molecular Biology, School of Basic Medical Sciences, Fudan University, Shanghai, China

**Keywords:** multi-allelic InDel, random DNA sequences, genome, mutation, multi-InDel

## Abstract

Multiple mutational events of insertion/deletion occurring at or around InDel sites could form multi-allelic InDels and multi-InDels (abbreviated as MM-InDels), while InDels with random DNA sequences could imply a unique mutation event at these loci. In this study, preliminary investigation of MM-InDels with random sequences was conducted using high-throughput phased data from the 1000 Genomes Project. A total of 3,599 multi-allelic InDels and 6,375 multi-InDels were filtered with multiple alleles. A vast majority of the obtained MM-InDels (85.59%) presented 3 alleles, which implies that only one secondary insertion or deletion mutation event occurred at these loci. The more frequent presence of two adjacent InDel loci was observed within 20 bp. MM-InDels with random sequences presented an uneven distribution across the genome and showed a correlation with InDels, SNPs, recombination rate, and GC content. The average allelic frequencies and prevalence of multi-allelic InDels and multi-InDels presented similar distribution patterns in different populations. Altogether, MM-InDels with random sequences can provide useful information for population resolution.

## 1 Introduction

Insertion/deletion polymorphisms (InDels) could be generated by the insertion or deletion of short DNA sequences and are thought to be bi-allelic (Bhangale et al., 2005). Currently, tremendous amounts of InDels in the human genome have been revealed through next-generation sequencing (Altshuler et al., 2010). According to the insertion or the deletion of DNA length and sequence types, InDels could be classified into insertions or deletions of single base pairs, expansions of monomeric base pairs or multi-base pairs of 2- to 15-bp repeat units, transposon insertions, and those containing apparently random DNA sequences (Mills et al., 2006). The formation of InDels could be attributable to slipped strand mispairing and a series of mechanisms such as cellular DNA repair against secondary structure, double-strand DNA break, defective mismatch, and unequal meiotic recombination (Ripley, 1982; Kaiser et al., 2007; Lieber, 2010; Bunting and Nussenzweig, 2013).

Generally, InDels could be caused by an insertion or a deletion mutation event in the genome across human history and are informative about human evolution and migration (Pereira et al., 2012). The expansions of short DNA sequences account for a large part of InDels, which might mainly be from polymerase slippage, as previous reports indicated a high mutation rate of polymerase slippage (Levinson and Gutman, 1987; Taylor et al., 2004; Montgomery et al., 2013). In contrast, InDels with random DNA sequences seem to rarely occur in the human genome since these InDels only account for a small part of InDels. Unlike duplication polymerase slippage, which can result in the multiple occurrences of gain or loss of repetitive units, InDels with random DNA sequences could imply a unique mutation event at these loci across human history (Montgomery et al., 2013).

Although the gain or loss of random DNA sequences rarely occurs, two or more such mutation events might happen to coincidentally occur at the same position in the genome, which could result in the birth of multi-allelic InDel loci with three or more variants (Auton et al., 2015). Additionally, the secondary mutation of insertion or deletion at the InDel locus might occur around the InDel locus, which could result in the generation of a multi-InDel locus, which was proposed to describe a set of InDels containing two or more InDel loci within a short fragment (Huang et al., 2014). In fact, multi-allelic single nucleotide polymorphisms (SNPs) and multi-nucleotide variants (MNVs) have been widely reported (Hodgkinson and Eyre-Walker, 2010; Harris and Nielsen, 2014; Prendergast et al., 2019). In contrast, multi-allelic InDel and multi-InDel loci have received relative lack of concern.

In this study, a collection of multi-allelic InDel markers and multi-InDel markers (abbreviated as MM-InDels) with random DNA sequences in the human genome was conducted using high-throughput sequencing phased data from the 1000 Genomes Project. MM-InDels with random sequences were investigated from aspects of genetic characteristics, genomic distribution, and comparison among worldwide populations.

## 2 Materials and Methods

### 2.1 Data Source

High-coverage whole-genome phased sequencing data were obtained from the working directory of the 1000 Genomes Project (Byrska-Bishop et al., 2021) (http://ftp.1000genomes.ebi.ac.uk/vol1/ftp/data_collections/1000G_2504_high_coverage/working/20201028_3202_phased/). The recombination rates (Rc) were based on the HapMap II combined map (International HapMap Consortium, 2003) (ftp://ftp.ncbi.nlm.nih.gov/hapmap/recombination/2011-01_phaseII_B37/genetic_map_HapMapII_GRCh37.tar.gz) and converted to Human Assembly (GRCh38/hg38) *via* the liftOver tool.

### 2.2 MM-InDel Marker Filtering

Phased sequencing data of a total of 2,504 unrelated individuals from the 1000 Genomes Project were utilized for the identification of MM-InDels in African ancestry population (AFR, *n* = 661), American ancestry population (AMR, *n* = 347), European ancestry population (EUR, *n* = 503), East Asian ancestry population (EAS, *n* = 504), and in South Asian ancestry population (SAS, *n* = 489).

The MM-InDels were filtered based on the following criteria: i) located on autosomes; ii) the insertion or deletion is a random sequence; iii) the flanking region of the insertion or deletion (from 12 bp upstream to 12 bp downstream) is a random sequence; iv) valid allele frequency >0.01 in the whole population; v) ≥3 valid alleles at a multi-allelic InDel marker or an multi-InDel marker; and vi) in a constructed multi-InDel marker, the largest physical interval between each two InDels is ≤20 bp.

Random sequences were determined by excluding expansions of monomeric base pairs or multi-base pairs of 2- to 15-bp repeat units. In the determination of the interval distance threshold in multi-InDel markers, the mean difference value of adjacent InDel marker numbers was calculated for S base pair distance as (*N*_s−2_ + *N*_s−1_)/2 − (*N*_s+1_ + *N*_s+2_)/2, where *N*_s_ implies the number of adjacent InDel marker with S base pair distance.

The physical position of the marker on the chromosome referred to Human Assembly (GRCh38/hg38). The VCFtools (Danecek et al., 2011) and homemade R and python scripts were applied to process the data for filtering of MM-InDels.

### 2.3 Statistical Analysis

Allele frequencies were calculated based on high-throughput sequencing data from the 1000 Genome Project using homemade python and R scripts. At each MM-InDel, alleles were nominated as alleles 1–6 in a descending order by allele frequency in the whole population. The GC contents were calculated based on the reference sequence of Human Assembly (GRCh38/hg38). The densities of SNPs, InDels, and MM-InDels were calculated into 100-kb bins based on high-throughput sequencing data from the 1000 Genome Project, while the density of recombination rate was calculated based on the HapMap II combined map. In order to visualize the commonalities and characteristics of the data, homemade R and python scripts were used for the construction of heatmap, histplot, Venn plot, and ideogram in this study.

## 3 Results

### 3.1 Multi-Allelic InDels With Random DNA Sequences in Human Genome

High-coverage sequencing phased data from the working directory of the 1000 Genomes Project were utilized to investigate InDel markers among worldwide populations. Of a total of 9,052,658 InDel markers, multiple alleles were observed in 364,170. Consequently, 3,599 multi-allelic InDel markers were filtered based on the criteria adopted in this study. The majority of these multi-allelic InDels was within 20 bp (91.90%), while the longest insertion or deletion sequence was 162 bp.

**FIGURE 1:**
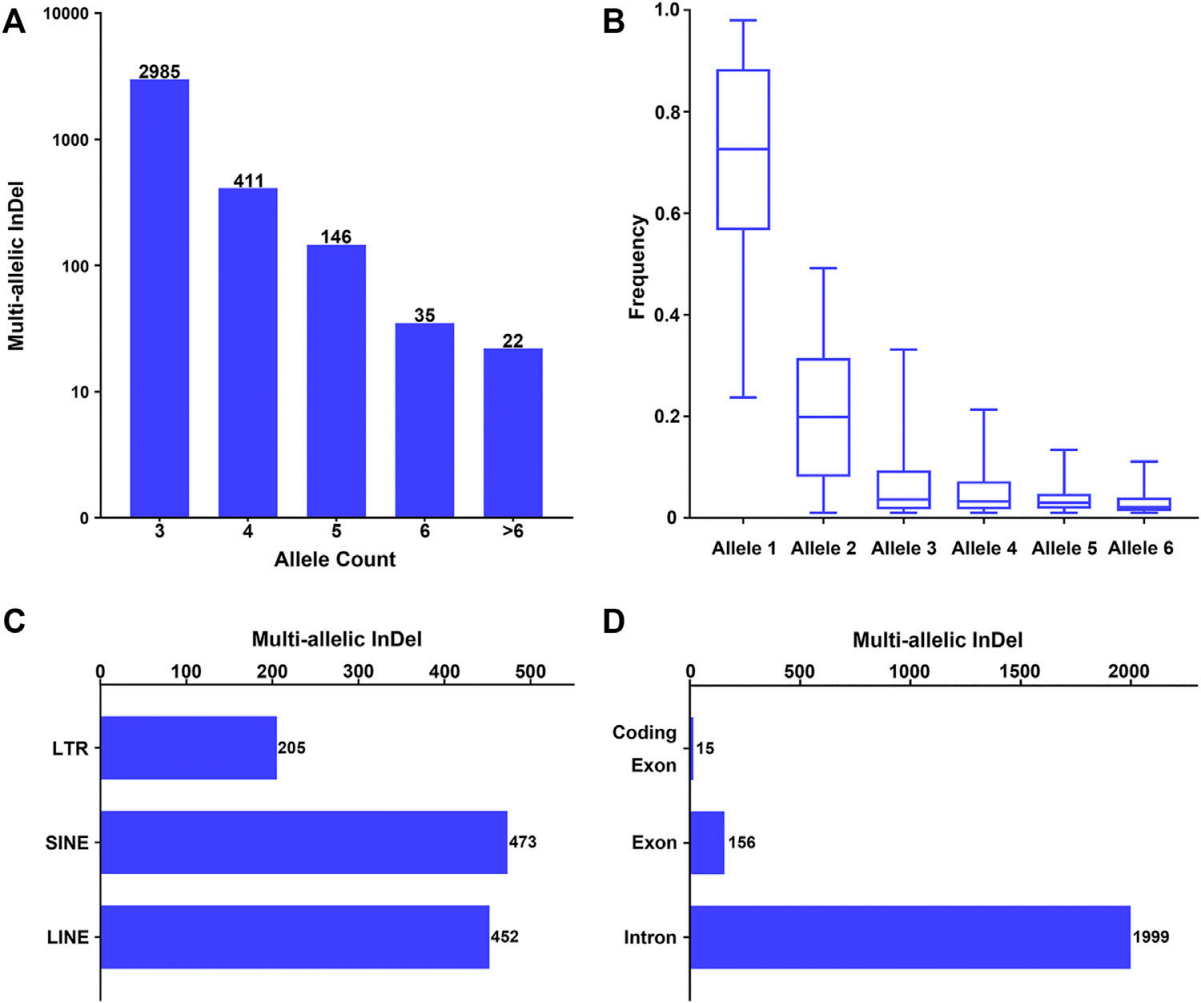
Investigation of multi-allelic InDels with random sequences across the genome. **(A)** Number of multi-allelic InDels with random sequences with relative allele counts. Alleles with allele frequency >0.01 were considered as valid alleles. **(B)** Frequency distribution of multiple alleles at the multi-allelic InDels. Alleles were sorted in a descending order by frequency in the global population at each multi-allelic InDel marker. Alleles with the 6 highest frequencies were referred to as alleles 1–6. **(C)** Genomic distribution of the multi-allelic InDels with random sequences located in repeat elements. *LTR*, long terminal repeat; *SINE*, short interspersed element; *LINE*, long interspersed element. **(D)** Genomic distribution of the multi-allelic InDels with random sequences located in annotated human genes.

Furthermore, these multi-allelic InDel markers were characterized based on the allele count, allele frequency, and physical position. As shown in Figure 1A, an allele count of 3 was observed in the majority of the candidate multi-allelic InDel markers (82.94%), while the number of multi-allelic InDels presented an exponential reduction with the increase in allele count. Besides, the allele frequencies at each multi-allelic InDel marker in the whole population were calculated, and the alleles were nominated as alleles 1–6 in a descending order by allele frequency. The overall allele frequency distribution of alleles 1–6 at these multi-allelic InDel markers is shown in Figure 1B. The results showed that the frequency of allele 1 ranged from 0.2370 to 0.9796, with a median frequency of 0.7266. The frequency of allele 2 ranged from 0.0102 to 0.4916, with a median frequency of 0.1989. The median frequencies of alleles 3–6 were 0.0365, 0.0325, 0.0300, and 0.0214, respectively. The overall frequency distribution of alleles 1–6 also demonstrated a similar exponential reduction (Figure 1B). Theoretically, the probability of the occurrence of a secondary mutation at an existing InDel marker would decrease with the increase of the cumulative number of mutational events.

The physical distribution of these multi-allelic InDels on autosomes was examined in relation to repeat elements and different genomic partitions. Accordingly, 1,128 multi-allelic InDels (31.34%) were located within known repeat elements, including short interspersed (SINE), long interspersed (LINE), and long terminal repeat (LTR) elements (Figure 1C). Meanwhile, 2,081 of the multi-allelic InDels (57.82%) were located within known genes (Figure 1D), in which 1,999 markers were located at the introns of genes, 156 markers were located in the exons of genes, and 15 multi-allelic InDels were located within the coding regions of genes (Supplementary Table S1).

### 3.2 Multi-InDels With Random DNA Sequences in Human Genome

**FIGURE 2:**
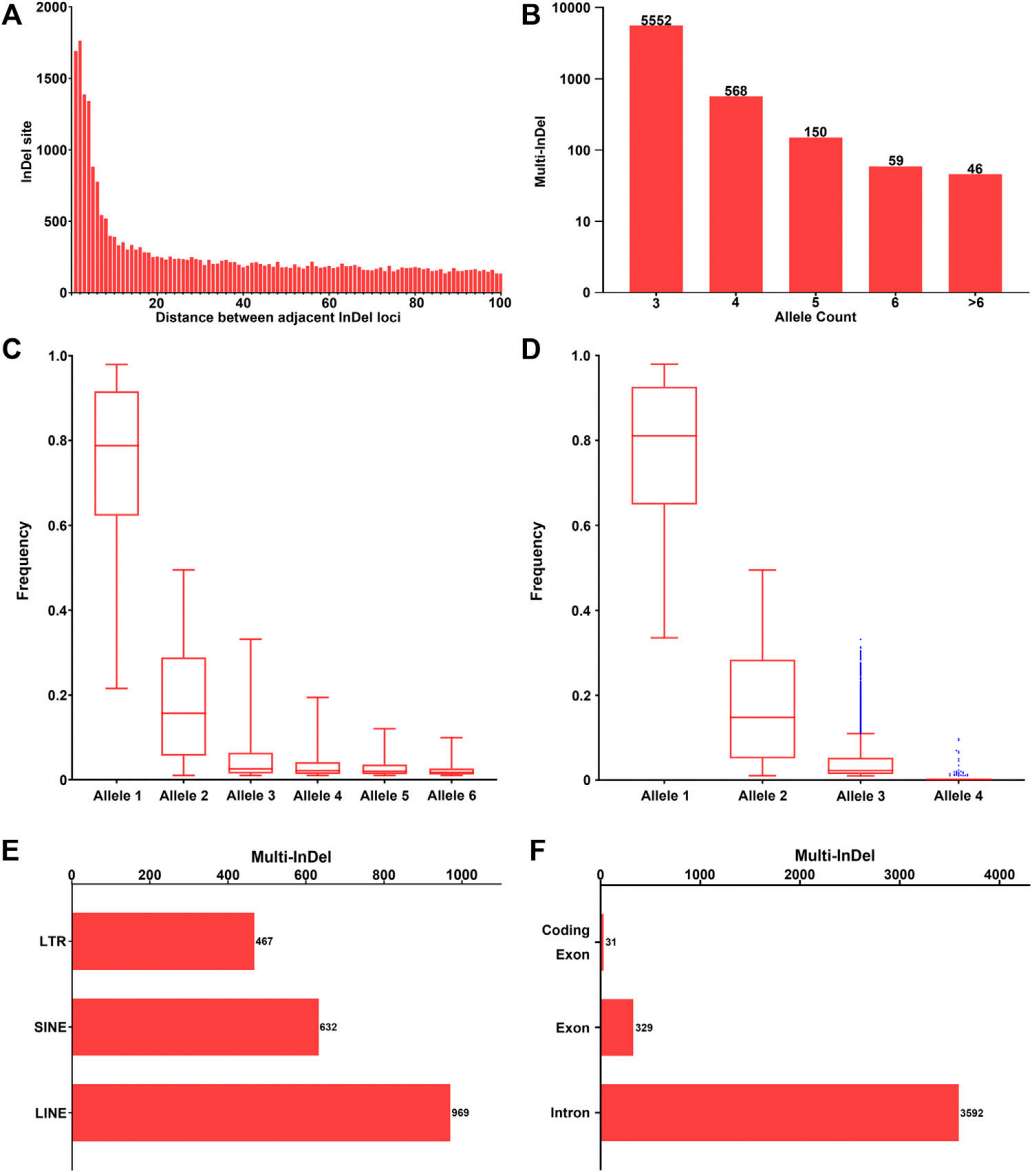
Investigation of multi-InDels with random sequences across the genome. **(A)** The number of sets of two adjacent InDels with random sequences with the interval 1–100 bp is shown. **(B)** Number of multi-InDels with random sequences with relative allele counts. Alleles with allele frequency ≥0.01 were considered as valid alleles. **(C)** Frequency distribution of multiple alleles at multi-InDels in worldwide populations. Alleles were sorted in a descending order by frequency in the global population at each multi-InDel marker. Alleles with the 6 highest frequencies were referred to as alleles 1–6. **(D)** Frequency distribution of multiple alleles at the multi-InDels consisting of two InDels. Alleles were sorted in a descending order by frequency at each multi-InDel marker. The *blue points* represent outliers of the distribution based on Tukey’s method. **(E)** Genomic distribution of the multi-InDels with random sequences located in repeat elements. *LTR*, long terminal repeat; *SINE*, short interspersed element; *LINE*, long interspersed element. **(F)** Genomic distribution of the multi-InDels with random sequences located in annotated human genes.

Investigation of the intervals between adjacent InDel loci with random DNA sequences was conducted across the genome. The more frequent presence of adjacent InDel loci was observed within a short distance, while the occurrence of multi-InDels remained relatively stable within a long distance (Figure 2A), which might indicate a potential relationship in the formation of InDel markers within short distances. Furthermore, the number of adjacent InDel loci decreased greatly within the 20-bp threshold, where the mean difference values were greater than 25. However, with the increase of the adjacent distance (>20 bp), the number of adjacent InDels stayed flat, with little change in the mean difference values. Therefore, in this study, we further characterized 6,375 multi-InDel markers with a limited interval ≤20 bp according to their allele (haplotype) count, allele frequency, and physical position on autosomes.

Generally, the number of included InDel loci in an multi-InDel marker ranged from 2 to 11 insertions or deletions. The length of the insertion or deletion sequence in a single InDel of multi-InDel markers was within 20 bp in 95.16% of loci, with a maximum of 144 bp. Most of these multi-InDel markers were observed with 3 alleles (87.09%), while their numbers decreased with the increase in allele count (Figure 2B). Regarding the frequency distribution of alleles 1–6 at multi-InDel markers, an exponential decreasing trend could be observed, which was similar to that observed in multi-allelic InDel markers (Figure 2C). Specifically, the frequency of allele 1 ranged from 0.2161 to 0.9792, with a median frequency of 0.7883. The median frequency of allele 2 was 0.1571, while the frequency of allele 2 ranged from 0.0104 to 0.4948. For alleles 3–6, the median frequencies were 0.0258, 0.0220, 0.0198, and 0.0184, respectively.

Moreover, the allele count and frequency distribution were analyzed in 4,681 multi-InDel markers that were composed of two bi-allelic InDel loci (Figure 2D). The results revealed that an allele count of 3 was commonly observed in 4,651 multi-InDel markers (99.36%), which supports the hypothesis that InDel mutations within a multi-InDel marker were formed based on secondary mutational events. Besides, those in multi-InDel markers with 4 alleles might result from chromosomal recombinations, rare insertion/deletion mutations, or typing errors in next-generation sequencing.

Meanwhile, the distribution of 6,375 multi-InDels on autosomes was investigated in relation to human genes and repeat elements, as well (Figures 2E,F). Subsequently, 2,059 multi-InDels (32.30%) were located within known repeat elements, including SINE, LINE, and LTR elements (Figure 2E). Besides, 3,904 multi-InDels (61.24%) were located within known genes (Figure 2F), in which 3,592 markers were detected at introns and 329 markers were located at exons. It was notable that 31 multi-InDels were observed at the coding regions of genes (Supplementary Table S2).

### 3.3 Distribution of MM-InDels With Random Sequences in Human Genome

**FIGURE 3:**
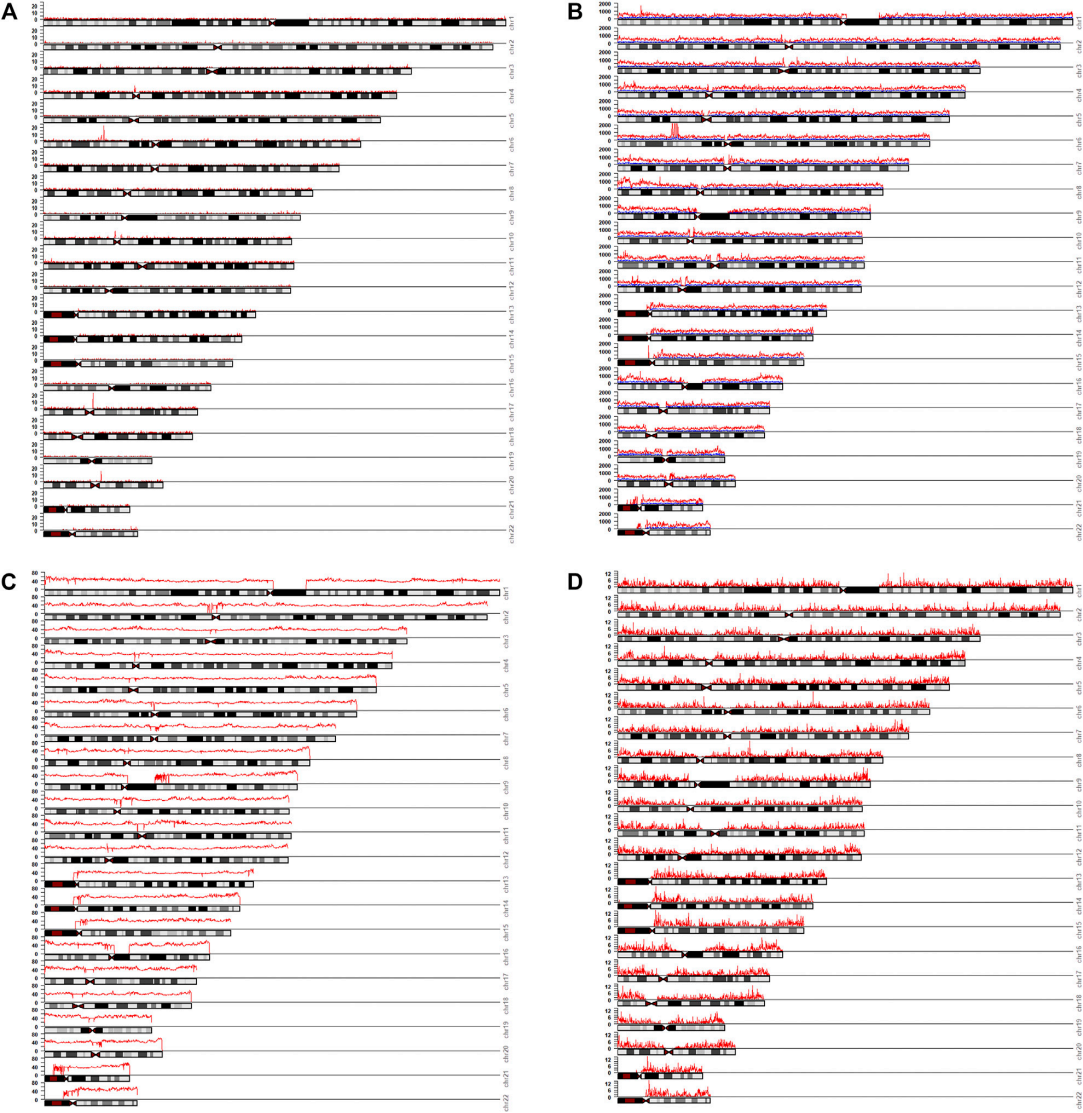
Distribution of MM-InDel markers and related factors across the genome. Distributions of MM-InDels with random sequences (in *red*) **(A)**, SNPs (in *red*) and InDels (in *blue*) **(B)**, GC contents (in *red*) **(C)**, and recombination rates (in *red*) **(D)** across the whole genome. The numbers of MM-InDels, all SNPs and InDels, and the average values of GC contents and recombination rates are shown for 100-kb bins.

The distribution of the selection of 9,974 MM-InDels (including 3,599 multi-allelic InDel markers and 6,375 multi-InDel markers) in the human genome was investigated. Generally, MM-InDels with random sequences showed an uneven and rare distribution on each autosome. The absence of markers presented in 71.12% of the regions across autosomes, specifically at the mitotic particle and short arm of autosomes (Figure 3A), which might be attributable to an underestimation of InDels or other polymorphic genetic markers due to the unsatisfactory quality of the current sequencing strategy. The density of MM-InDels per 100 kb appeared to be ≤3 at most of the other regions (28.66%; Figure 3A). Meanwhile, the enrichment of MM-InDels has been observed with the occurrence of ≥10 markers per 100 kb, which were at chr4: 49.1–49.2 Mb (*n* = 10), chr6: 32.5–32.8 Mb (*n* = 10–22), chr10: 38.5–38.9 Mb (*n* = 10–11), chr17: 26.8–26.9 Mb (*n* = 23), and chr20: 31.1–31.2 Mb (*n* = 16), which might imply high dynamics of these chromosomal regions.

**FIGURE 4:**
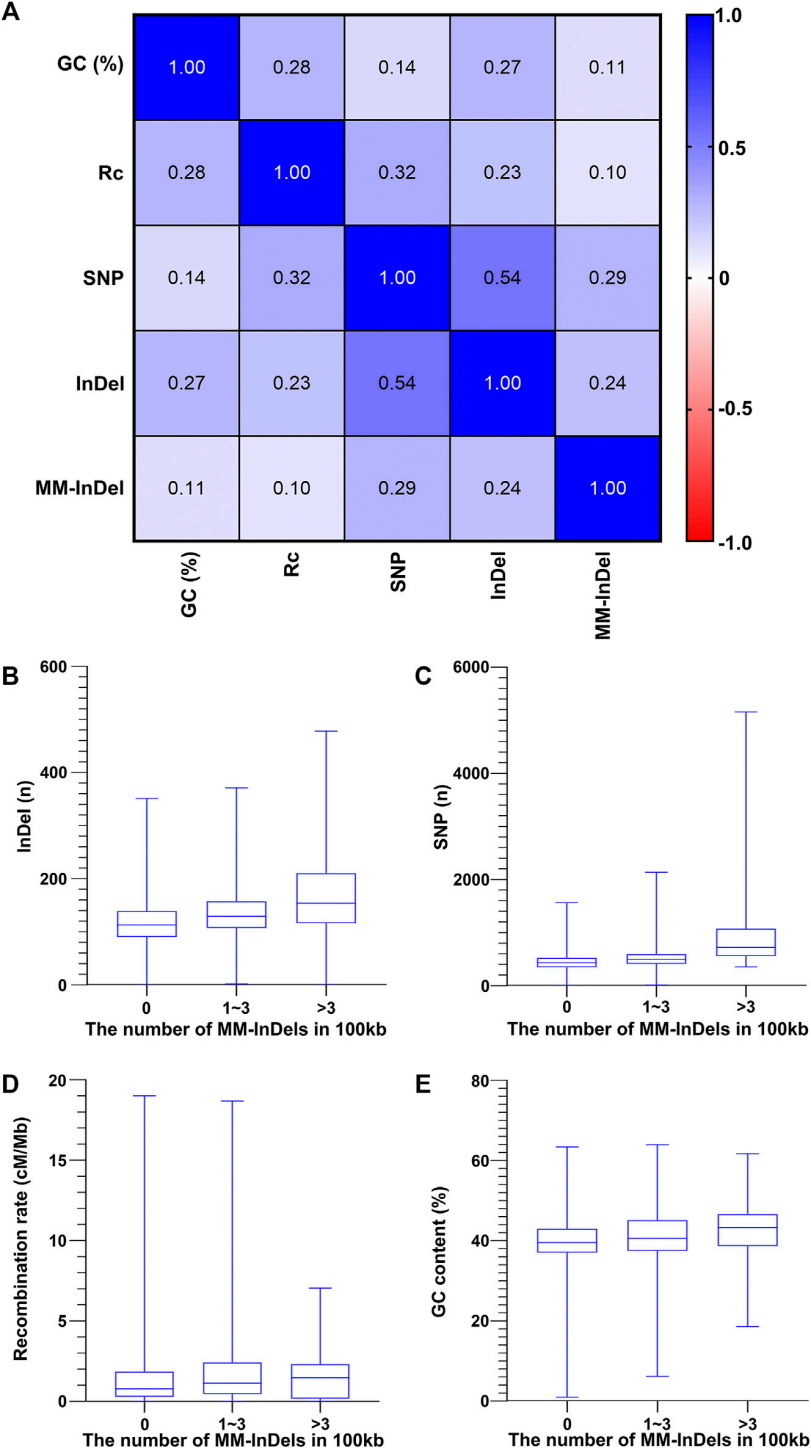
Relationship between the occurrence of MM-InDel markers with random sequences and different genomic factors. **(A)** Correlation analysis between the density of MM-InDels with random sequences and genomic factors such as the density of SNPs, the density of InDels, GC contents [GC (%)], and recombination rates (Rc). The average value for 100-kb bins is utilized. *p*-values were all <0.0001. **(B–E)** Correlation of MM-InDels harboring random sequences with the density distribution of InDels **(B)**, SNPs **(C)**, GC content **(D)**, and recombination rate **(E)**.

In order to understand the potential cause in the shaping of MM-InDel generation across the whole genome, the marker densities of both single InDels and SNPs were analyzed in the same scale as the MM-InDels (Figure 3B), and the average recombination rate and GC content were also calculated for 100-kb bins (Figures 3C,D). The relationship between the generation of MM-InDels and the presence of InDels and SNPs, recombination rate, and GC content was investigated (Figure 4A). The results showed a moderate correlation between the distribution of MM-InDels and the presence of InDels and SNPs (*r* = 0.24 and 0.29, *p* < 0.0001), while the recombination rate and GC content demonstrated a weak correlation with MM-InDel distribution (*r* = 0.10 and 0.11, *p* < 0.0001).

Additionally, each potential factor was further inspected within different groups of MM-InDel densities per 100 kb on autosomes. As shown in Figures 4B–E, the average density of InDels and SNPs along with the recombination rate and GC content appeared relatively lower at regions with the absence of MM-InDels. In contrast, in those regions that contained 1–3 or >3 MM-InDels, an increase of the average magnitude could be observed. Overall, there might be a potential causal effect between the shaping of MM-InDels with random sequences and other genetic markers, including InDels and SNPs, while higher recombination rate and GC content might be favorable for the formation of MM-InDels.

### 3.4 Characteristics of MM-InDel Markers Among Different Populations

**FIGURE 5:**
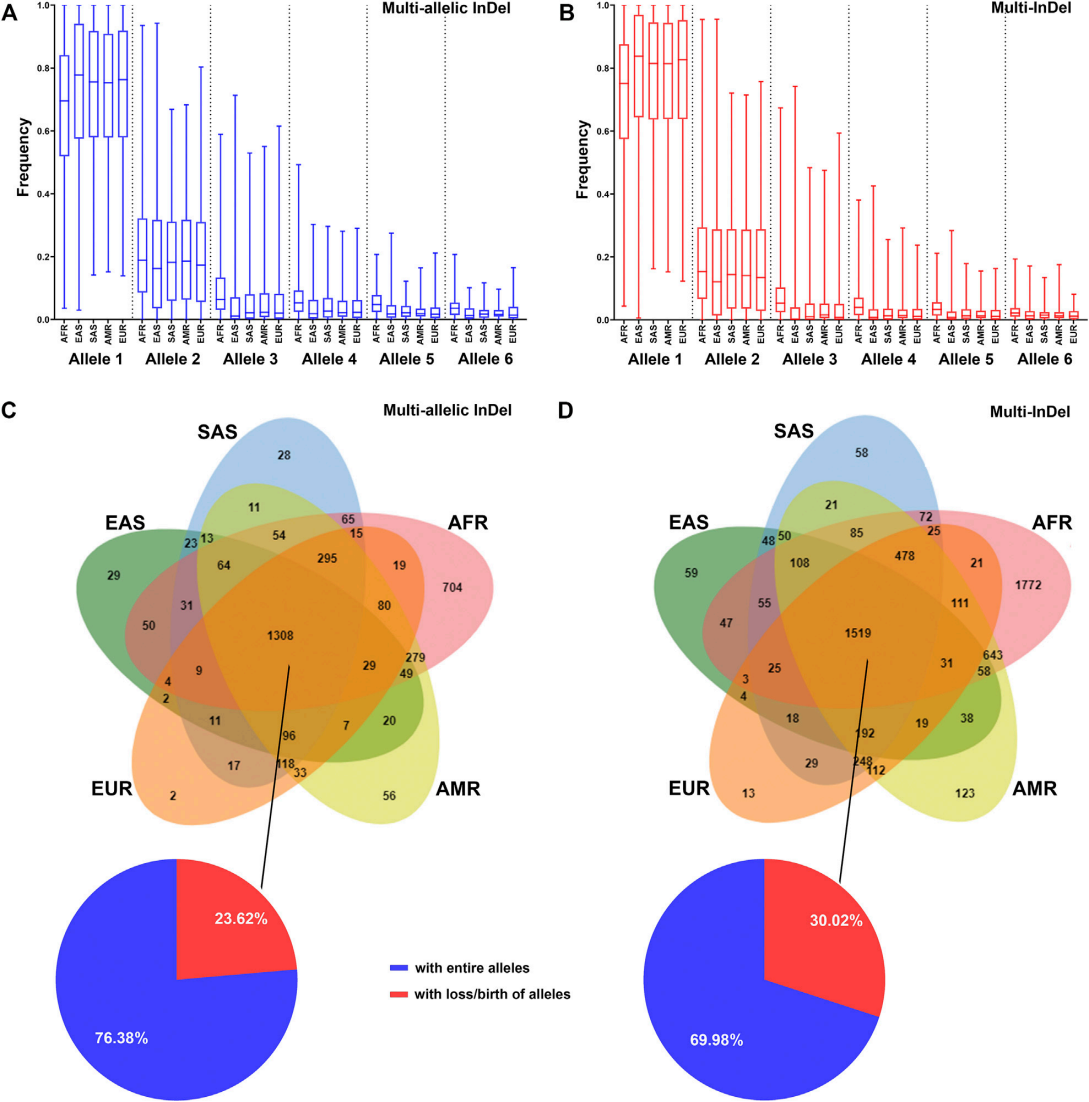
Distribution of MM-InDel markers in worldwide populations. **(A,B)** Allele frequency distributions of multi-allelic InDels with random sequences **(A)** and multi-InDels with random sequences **(B)** among the African ancestry population (*AFR*), American ancestry population (*AMR*), European ancestry population (*EUR*), East Asian ancestry population (*EAS*), and South Asian ancestry population (*SAS*). Alleles were sorted in a descending order by frequency at each marker in the total population. **(C,D)** Venn diagram showing the distributions of multi-allelic InDels with random sequences **(C)** and multi-InDels with random sequences **(D)** in different population cohorts. The overlapping region indicates the number of MM-InDels with three or more alleles. The pie plot indicates the proportions of MM-InDels with entire alleles (*blue*) or with loss/birth of alleles (*red*) in the populations.

The allelic frequency of MM-InDels was investigated in different populations. In a total of 9,974 MM-InDels, the average frequency of the most observed alleles ranged from 0.67 to 0.74 for multi-allelic InDels (Figure 5A) and from 0.71 to 0.78 for multi-InDels (Figure 5B) among all populations. For the second most common alleles, the average frequency ranged from 0.20 to 0.22 for multi-allelic InDels and from 0.17 to 0.20 for multi-InDels. Similar distribution patterns of the average allelic frequencies of multi-allelic InDels and multi-InDels could be observed in different populations (Figures 5A,B). The African population presented the highest average frequency of the third and more infrequent alleles, while the East Asian population showed the opposite pattern for multi-allelic InDels and multi-InDels.

The allele prevalence of MM-InDels was subsequently inspected among worldwide populations. As shown in Figures 5C,D, 1,308 multi-allelic InDel markers and 1,519 multi-InDel markers commonly presented multiple alleles in all five populations. Among these sites, 999 multi-allelic InDels (76.38%) and 1,063 multi-InDels (69.98%) exhibited entire alleles in all five populations, although the allelic frequencies appeared different in these five populations, which implies an ancient formation of these MM-InDels. In contrast, the loss or birth of alleles in these five populations occurred at multi-allelic InDels (23.62%) and multi-InDels (30.02%), which could imply a population differentiation. Furthermore, single population-specific alleles were observed at 819 multi-allelic InDels (22.76%) and 2,025 multi-InDels (31.76%), with the African population-specific ones accounting for 19.56% at multi-allelic InDels and 27.80% at multi-InDels.

## 4 Discussion

A total of 9,974 MM-InDels with random DNA sequences of insertion or deletion were filtered, and the occurrence of MM-InDels with random sequences was further characterized. In fact, there should be much more MM-InDels with random DNA sequences that can be observed when lowering the filtering threshold of allele frequency. For the generation of MM-InDels with random sequences, the more frequent presence of adjacent InDels was observed within 20 bp (Figure 2A), while most InDels within multi-InDel markers were also under 20 bp (95.16%). In fact, the enrichment of neighboring polymorphisms has been reported in the human genome (Hodgkinson and Eyre-Walker, 2010), which is in line with our observation. A correlation of the separate mutational events forming MM-InDels might be assumed in a closer distance with shorter mutational sequence lengths.

Similar to the formation of SNPs in adjacent regions, the insertion/deletion mutations *in situ* or adjacent to another InDel locus could either be in a sequential order or in a single generation (Prendergast et al., 2019). In this study, the investigation of MM-InDels with random sequences was further conducted to inspect the sequential order of mutations that formed multiple alleles by limiting the allele count to ≥3 in the filtering of MM-InDels. MM-InDel loci with three alleles accounted for 85.59% of the obtained candidates. Additionally, previous studies suggested that MM-InDels containing two InDels have three instead of four alleles (Huang et al., 2014; Li et al., 2021), which is in line with our observation. Since it is difficult for multi-allelic InDels to repeatedly mutate into an identical allele and multi-InDels with two InDel loci presented a tri-allelic pattern in the population, a secondary mutation event could be inferred at these loci. Furthermore, a small number of multi-allelic InDels and multi-InDels with ≥3 InDel loci within 20 bp presented four or more alleles in the population, which implies that multiple mutation events still occur at these loci even though InDels with random DNA sequences have a potentially low mutation rate. In fact, an exponential decrease in the total number of markers was presented in both multi-allelic InDels and multi-InDels with the increase in allele counts, while the biased frequencies observed in each allele in these MM-InDels also demonstrated a similar decrease pattern.

The density of InDel markers was reported to be related to the existence of SNPs or the GC content (Tian et al., 2008; Zhang et al., 2008; Miles et al., 2016). Moreover, the correlation between the generation of InDels and recombination rate was studied in the previous study, which indicated that the occurrence of InDels might be attributed to homologous recombination (Kvikstad et al., 2009; Rao et al., 2010; Zhu et al., 2019). In this study, the distribution and shaping of MM-InDels were explored with reference to the existence of InDels, SNPs, the GC content, and the recombination rate in different regions along each chromosome. Similar to SNPs and InDel markers, the distribution of MM-InDels also showed an uneven pattern, while several large hotspots and many small hotspots were observed throughout the genome. The recombination and mutation hotspots were related to GC-rich loci, while fork stalling and the template switching mechanism could cause DNA repair machinery and mutation formation (Nesta et al., 2021). The generation of MM-InDels with random sequences might be attributable to recombination and DNA secondary structure repair (Montgomery et al., 2013). Therefore, the moderate correlation of MM-InDel density with the GC content and recombination rate might be partially due to the mechanism of the generation of MM-InDels. Additionally, most of the MM-InDels were located nearby repeat elements, which might have contributed to the generation of MM-InDels by virtue of non-homologous recombination (Szafranski et al., 2019).

As indicated in previous studies, the incidence of InDel markers might have great impact on the gene structure and evolution of gene expression (Kaiser et al., 2007; Kloosterman et al., 2015; Shendure and Akey, 2015). Relative to amino acid replacements or nonsense mutations caused by SNPs, InDels in the coding exon always cause the change of open reading frame (ORF) and amino acid. In this study, we observed almost 50% of MM-InDels associated with annotated human genes and 100 of the MM-InDels directly located at the coding exon. Although gene-related sequences account for approximately 40% in the whole human genome (Piovesan et al., 2019), MM-InDels with random sequences appeared to be enriched and related to gene expression, which might be due to the higher probability of gene sequence attack and recombination during gene expression and replication in the G1 phase. Meanwhile, in repeat elements such as LINE and SINE, MM-InDels will affect the generation and variation of InDel in other parts of the genome due to homologous and non-homologous recombination.

Investigation of the collection of MM-InDels demonstrated both consistency and differentiations among worldwide populations. Most of the MM-InDels with random sequences were observed in more than one population, while approximately 28.34% of MM-InDels were common in different populations, in which the same alleles could be assumed to have been formed by secondary mutations at a very ancient time. In the meantime, the presence or absence of the third allele of MM-InDels among different populations might depend on the time point of secondary mutations during diachronic population migration and expansion. The diversities of the distribution of allele frequency might also be affected by the founder effect. Among all the studied populations, the AFR possessed the majority of the population-unique MM-InDels with a relatively higher average frequency of the minor allele, prompting more ancient mutation events in this population. These phenomena are in line with the law of population migration and the fact that humans originated in Africa (Bergström et al., 2021). The divergence of the MM-InDels in the scale of worldwide populations might also provide a fine resolution of population differences. Additionally, the highly consistent characteristics of the distribution between the multi-allelic InDels and multi-InDels among worldwide populations would indicate similar mutational events in the generation of the MM-InDels, where secondary insertions or deletions took place either *in situ* or adjacent to the progenitor InDel locus.

## Data Availability

The original contributions presented in the study are included in the article/Supplementary Material. Further inquiries can be directed to the corresponding author.
